# Using Big Data to Monitor the Introduction and Spread of Chikungunya, Europe, 2017

**DOI:** 10.3201/eid2506.180138

**Published:** 2019-06

**Authors:** Joacim Rocklöv, Yesim Tozan, Aditya Ramadona, Maquines O. Sewe, Bertrand Sudre, Jon Garrido, Chiara Bellegarde de Saint Lary, Wolfgang Lohr, Jan C. Semenza

**Affiliations:** Umeå University, Umeå, Sweden (J. Rocklöv, A. Ramadona, M.O. Sewe, W. Lohr);; New York University, New York, New York, USA (Y. Tozan);; European Centre for Disease Prevention and Control, Stockholm, Sweden (B. Sudre, J. Garrido, C.B. de Saint Lary, J.C. Semenza)

**Keywords:** big data, data science, chikungunya, arbovirus, *Aedes albopictus*, Europe, human mobility, social media, vectorial capacity, vector-borne infections, viruses

## Abstract

With regard to fully harvesting the potential of big data, public health lags behind other fields. To determine this potential, we applied big data (air passenger volume from international areas with active chikungunya transmission, Twitter data, and vectorial capacity estimates of *Aedes albopictus* mosquitoes) to the 2017 chikungunya outbreaks in Europe to assess the risks for virus transmission, virus importation, and short-range dispersion from the outbreak foci. We found that indicators based on voluminous and velocious data can help identify virus dispersion from outbreak foci and that vector abundance and vectorial capacity estimates can provide information on local climate suitability for mosquitoborne outbreaks. In contrast, more established indicators based on Wikipedia and Google Trends search strings were less timely. We found that a combination of novel and disparate datasets can be used in real time to prevent and control emerging and reemerging infectious diseases.

Many sectors of society have taken full advantage of new opportunities provided by big data, but public health has not (*1*). Although electronic health records have long been used in surveillance, novel applications of big data are rare. Internet search query data from Google or Wikipedia have been applied to anticipate influenza epidemics but are hampered by several limitations, including specificity and granularity (*2*–*4*). More recently, crowdsourcing of symptoms through emails, text messages, or tweets has been explored, and outbreaks have been tracked by scanning high-volume surveillance systems (*5*,*6*). However, when it comes to fully harvesting the potential of big data, public health still lags behind other fields. Using chikungunya as a case study, we illustrate how big data can help tackle emerging infectious diseases through prevention, detection, and response.

A key driver of the emergence and spread of vectorborne diseases is human mobility (*7*–*10*), yet little is known about the epidemiologic consequences of mobility patterns at different spatial scales within the context of vectorborne diseases. A main obstacle to studying the complex interactions between human hosts, pathogens, and vectors has been the limited availability of spatiotemporal datasets for analyzing human mobility patterns. Prior research relied on low-resolution mobile phone records, such as call and messaging logs from mobile phone networks (*11*–*13*), for which biases were notable (*14*,*15*). Furthermore, use of mobile phone data for tracking human mobility is likely to be fraught with privacy concerns and data access restrictions (*15*).

Recently, social media has emerged as an alternative source of real-time, high-resolution geospatial data on a large scale (*1*,*15*). Use of this unique aspect of publicly available social media data to study the human dimensions of the introduction and spread of emerging infectious diseases has not been explored to its fullest extent. In areas where risk for virus importation and onward transmission is heightened, such knowledge can inform outbreak preparedness and response planning by pinpointing receptive areas where proactive countermeasures should be implemented in a timely fashion (*16*,*17*).

The impediments to using big data in public health are not only the size of the databases but also the complexity of their processing. The challenges include 3 main dimensions: volume, velocity, and variety (*18*–*20*). Volume calls for statistical sampling; velocity, for instant access to near real-time transaction data; and variety, for management of nonaligned data structures. We illustrate how big data can be used to monitor the introduction and spread of the 2017 chikungunya outbreak in Europe by tackling these challenges (*18*–*20*).

To assess risk for virus importation from international areas with active chikungunya transmission, we extracted air passenger volume from large-scale aviation data. To quantify the risk for short-range dispersion (defined as the potential for onward transmission and spread of chikungunya virus from the initial outbreak foci to other areas during transmission season), we used a mining algorithm to process quasi–real-time, geolocated Twitter activity data and computed mobility patterns of users. We have previously shown that mobility data from Twitter users is predictive of disease spread (*21*). We then estimated the seasonal vectorial capacity of *Aedes albopictus* mosquitoes to transmit chikungunya virus and linked it with human mobility patterns. We further complemented these data with Internet and information search activities related to chikungunya infection, vectors, and clinical signs and symptoms collected from Wikipedia and Google Trends. Last, we estimated the empirical basic reproduction number (R_0_) from the outbreaks and compared these numbers with our model predictions of epidemic potential based on climate conditions. More detail on our methods in Appendix 1. 

## Climate Suitability: Vectorial Capacity

**Figure 1:**
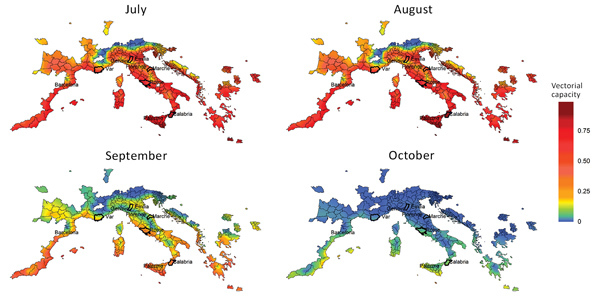
Vectorial capacity estimates based on average temperature conditions in Europe with stable populations of *Aedes albopictus* mosquitoes around chikungunya outbreak zones, Italy and France, July–October 2017. Heavy outlines indicate the outbreak areas. The vectorial capacity translates to an average basic reproduction number in the range of 2–3 in Anzio and Rome and in the range of 3–4 in Calabria during the months of July and August for an infectious period of 4 days.

**Figure 2:**
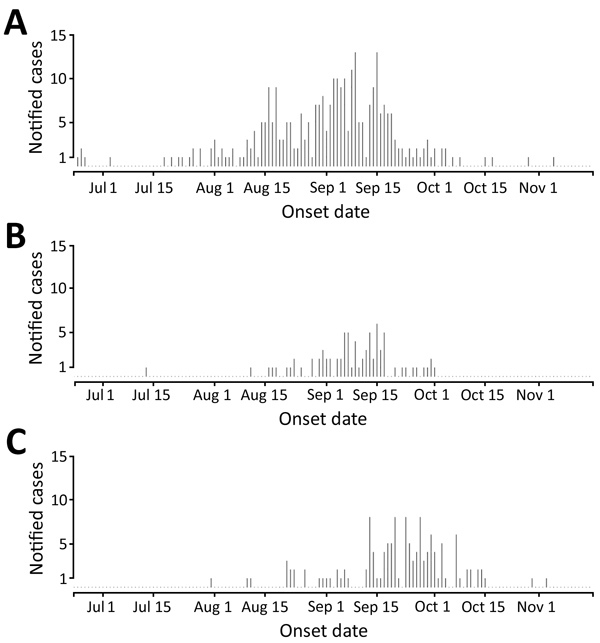
Notified chikungunya cases in the Anzio (A), Rome (B), and Calabria (C) regions and basic reproduction number (R_0_) estimates of outbreaks, June–October 2017, Italy.

The vectorial capacity of *Ae. albopictus* mosquitoes to transmit chikungunya virus in areas of Europe where the vector is established (*17*), such as the outbreak zones in France and Italy, was estimated to be high in July and August but lower in September and October. Estimates of suitability were low in October for most areas, except those in southern Italy and Greece and southeastern Spain (Figure 1). Overall, warmer than average temperatures led to a substantial increase in vectorial capacity during the study period (June–October 2017) (Appendix 2 Figure 1). Using empirical data from the outbreaks in Italy (*22*), we estimated R_0_ to be 2.28 (95% CI 2.01–2.59) for the Anzio region, 3.54 (95% CI 2.62–4.97) for the Rome region, and 3.11 (95% CI 2.16–4.79) for the Calabria region (Figure 2).

## Long-Range Importation: Air Passenger Volume

**Figure 3:**
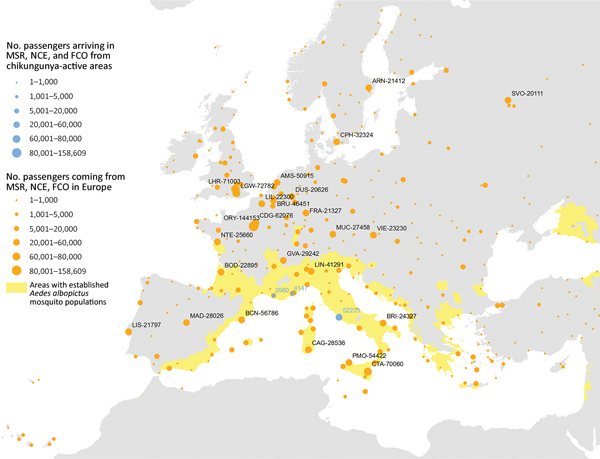
Incoming passengers from chikungunya active transmission areas and outgoing passengers to other airports in Europe from Rome (FCO), Marseille (MRS), and Nice (NCE) airports, August 2017. The stable vector presence area is highlighted in yellow.

**Figure 4:**
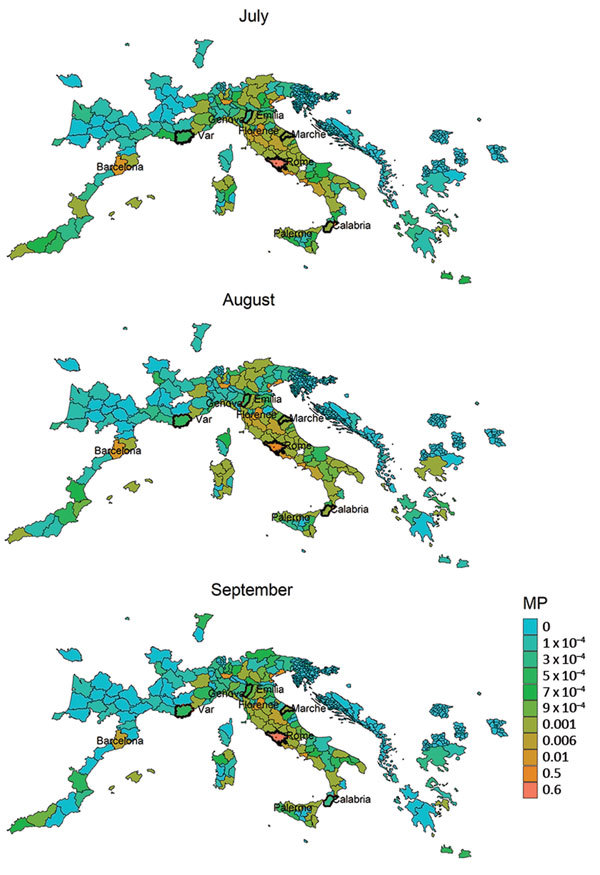
MP estimates from the Lazio region, Italy, to areas in Europe with stable populations of *Aedes albopictus* mosquitoes, July–September 2017. Heavy outlines indicate the chikungunya outbreak areas. MP, mobility proximity.

On average, ≈50,000 air passenger-journeys (1 passenger flight, including all legs of travel) were taken each month from areas with active chikungunya transmission worldwide to the outbreak zones (Figure 3). Specifically, in August, 56,300 passengers from outbreak zones were estimated to arrive in Rome, 6,484 in Nice, and 5,629 in Marseille. The passenger-journey volume into Europe when the outbreak started in June is shown in Appendix 2 Figure 2. The countries with the highest number of departing passengers in August were Thailand (352,332 passengers), Brazil (255,439 passengers), and India (301,298 passengers). According to molecular epidemiology, the genome sequence of a chikungunya virus isolate from the Lazio region of Italy revealed the East/Central/South African lineage, Indian Ocean sublineage, which is similar to that of recent sequences from Pakistan and India (*23*). We also extracted air passenger-journey data for flights from the outbreak zones in southeastern France and central Italy to other areas in Europe (Figure 3). The top 5 destinations with the highest volume were the larger metropolitan areas of Europe, most of which were outside the boundaries of areas where the vector is known to be present (Figure 4). However, high flight connectivity was observed from the outbreak zones to Barcelona (Spain) and Catania and Palermo (Italy).

## Short-Range Dispersion: Geocoded Tweets

The spatiotemporal analysis of geocoded Twitter data showed strong human mobility from Lazio (Figure 4) and the Var department in France (Appendix 2 Figure 3) toward several larger cities where *Ae. albopictus* mosquitoes are present. The top 10 estimates of mobility out of the 2 outbreak zones of Var and Lazio showed the strongest pattern for potential dispersion of chikungunya virus not only into the areas geographically close to the outbreak zones but also to several relatively large cities in Italy, France, and Spain (Table). The monthly mobility patterns during the study period varied between months; for example, the vacation month of August showed a stronger mobility pattern out of Var to areas not in direct connectivity, most notably to Rome (Appendix 2 Figure 4). When we contrasted the mobility proximities between the 2 outbreak zones, we observed the highest proximities within countries (Figure 4; Appendix 2 Figure 3). Although the Var and Lazio outbreak zones experienced high mobility proximity to Barcelona, Lazio was also highly connected to southern Italy (e.g., Catania and Palermo), in close proximity to the chikungunya outbreak in the Calabria area, which was also observed in the International Air Transport Association (IATA) flight passenger data (Figures 3, 4). In Italy, cases were first notified in Anzio at the end of June, followed by notifications in Rome later in July, and in Calabria in early August in order of temporal appearance (Figure 2). In our mobility analysis, we identified the mobility links to all outbreak regions (Figure 4), with the exception of the Emilia-Romagna region, although the region neighboring Emilia-Romagna was positive in our analysis. The mobility patterns correlated more strongly to the outbreak regions in July and August.

**Table:** Top 10 areas where mobility proximity to the 2 chikungunya outbreak zones was highest, Europe, August 2017

Rank	Southern Europe			Lazio region	
	From Var department, France	From Lazio region, Italy		From Anzio	From Rome
1	Alpes-Maritimes	Florence		Roma	Vatican
2	Bouches-du-Rhône	Milano		Nettuno	Fiumicino
3	Torino	Napoli		Sabaudia	Sabaudia
4	Paris	Venezia		Ardea	Civitavecchia
5	Alpes-de-Haute-Provence	Paris		Civitavecchia	Santa Marinella
6	Rhone	Barcelona		Pomezia	Tivoli
7	Hérault	Perugia		Aprilia	Anzio
8	Vaucluse	Latina		Cisterna Di Latina	Ladispoli
9	Barcelona	Siena		Fondi	Pomezia
10	Baeleares	Salerno		Amatrice	Valmontone

**Figure 5:**
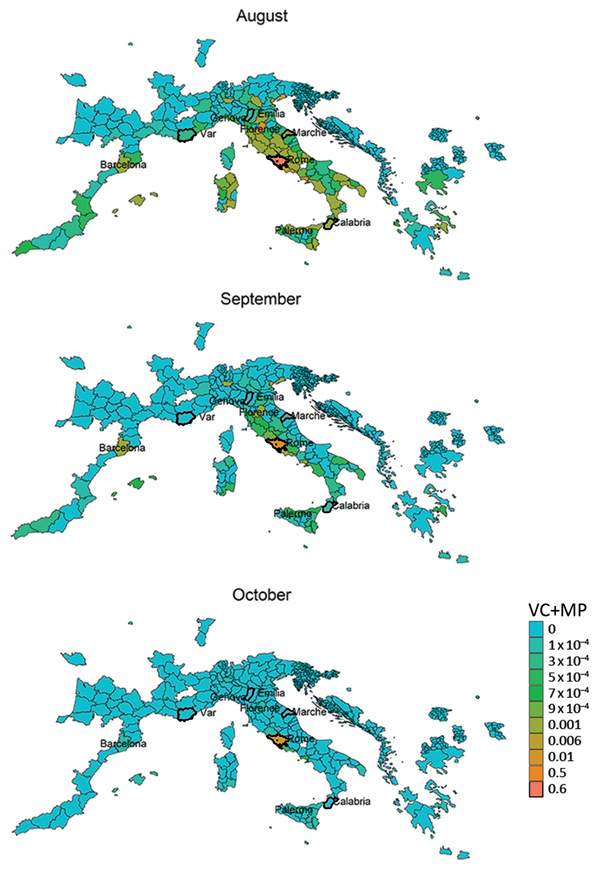
Estimated areas of risk for chikungunya spread from the outbreak areas of Anzio and Rome in the Lazio region, Italy, based on combined VC and estimates, August–October 2017. Heavy outlines indicate the outbreak areas. MP, mobility proximity; VC, vectorial capacity.

A closer look at the Lazio outbreak zone in Italy revealed strong connectivity between Anzio (where the first cases in Italy were confirmed) and Rome (where a higher number of cases were notified) (Figure 5). We compiled the top 10 mobility proximity areas from the outbreak zones of Anzio and Rome in August and September (Table). Although the highest mobility proximity from Anzio was to Rome in August and September, the mobility proximity from Rome to Anzio was also found among the top 10 destinations. Overall, Rome had higher connectivity to many more areas than Anzio.

## Synergistic Effects: Human Mobility and Transmission Suitability

**Figure 6:**
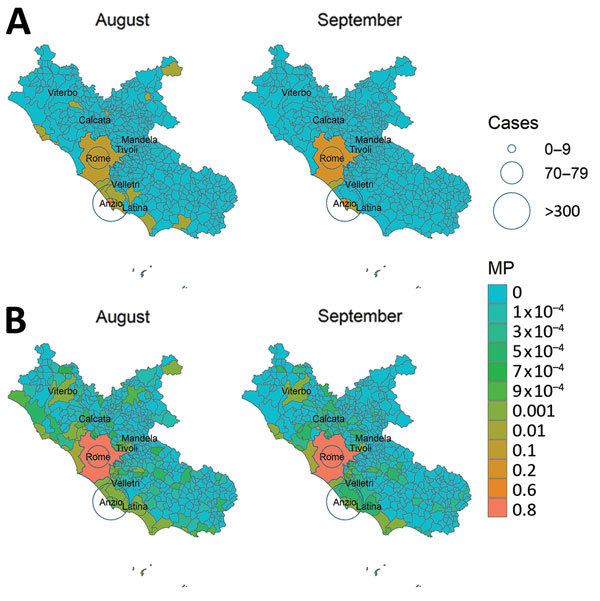
Estimated areas of risk for chikungunya spread from the outbreak areas in Lazio region, Italy, based on MP estimates, August–September 2017. A) Anzio; B) Rome. Circles indicate number of reported cases. MP, mobility proximity.

We derived risk maps for autochthonous chikungunya transmission by combining the vectorial capacity and mobility proximity estimates for the Lazio region in Italy and Var department in France for August–October 2017 (Appendix 2 Figure 4). The areas at risk because of the outbreak in Var were identified to be located along the French and northern Spanish Mediterranean coastlines, Mallorca, and Rome in August (Appendix 2 Figure 4); the risk regions for the Lazio outbreak in August included large parts of Italy as well as areas in France, Spain, and Greece (Figure 6). In general, the size of the area at risk contracted in September and more so in October because of less favorable climate conditions, except in the most southern region of Italy (Figure 6), such as the Calabria region, where the outbreak also empirically continued longer in the fall (Figure 2).

In the Lazio region, an analysis of the combination of vectorial capacity (Appendix 2 Figure 5) and mobility proximity revealed a higher transmission potential in August (Appendix 2 Figure 6), with implications for targeting surveillance and outbreak control activities to this region. The largest area of risk for spread from Anzio was Rome, but the risk for spread from Rome was more widespread in the region (Appendix 2 Figures 6, 7). The areas at risk for spread in the Lazio region differed during August compared with September and October.

## Wikipedia and Google Trend Indicators

For the outbreaks in Italy, several pathogen and vector-related Wikipedia and Google Trend search pattern anomalies are illustrated (Appendix 2 Figure 8). The peaks in these abnormalities coincided with the peak of the outbreak and therefore are not useful for early detection and response activities. Detailed information about Wikipedia and Google Trend indicators are provided in Appendix 3.

## Big Data and Emerging Infectious Diseases

In light of the arrival and explosive expansion of chikungunya in the Americas in 2013 through *Ae. aegypti* moquitoes (*24*), big data offer the opportunity to monitor the introduction and spread of chikungunya in Europe. An outbreak can be divided, broadly speaking, into 2 distinct phases. The first phase is importation of the virus via a viremic person into a virus-naive population. For this phase, we used big data (volume) to estimate air passenger-journeys from areas with active chikungunya transmission as a measure of the force of introduction of the virus into the outbreak zones in Europe. To identify areas with onward transmission risk, we also considered the volume of air passengers leaving these outbreak zones. For the second phase, the establishment of autochthonous transmission in Europe is a function of virus importation, population density, vector activity, climate conditions, exposure patterns, and several other factors that are more difficult to quantify (*17*). Our study addressed some of these epidemiologic challenges by using big data. Rather than a Twitter content analysis, which has been performed for several outbreaks (*25*–*28*), we used near–real-time geocoded Twitter data (velocity) to quantify human mobility patterns and disentangled connectivity between populations. Mobility estimates also reflect population density and indirectly take into account exposure patterns because such populations on the move are occasionally susceptible to exposure and are also a source of exposure. The ecology of the virus and the human-vector transmission cycle were captured by vectorial capacity (variety), which quantified transmission risk on the basis of climate conditions. Thus, we were able to quantify the trajectory of an arbovirus outbreak by dissecting and better understanding its phases.

Our analysis of big data revealed distinct mobility patterns between the outbreak zones in France and Italy, between Rome and Anzio, and between Rome and most of the local outbreak clusters in Italy. However, the potential effects of these mobility patterns on local spread need to be confirmed epidemiologically by phylogenetic analyses. Although the sensitivity of our risk maps based on mobility and climate data to identify areas at risk for virus spread was good, the specificity needs to be further improved, for example, by including local contextual factors such as land use and vector activity. Wikipedia page hits and Google Trends have been proposed as resources for disease surveillance and outbreak detection. However, our analysis demonstrates that these sources seemed to mainly indicate public awareness of the chikungunya outbreaks as they peaked. For such reasons, they seem to be of little use for early response.

The combination of short-distance air passenger-journeys (within Europe, as opposed to overseas) and geocoded Twitter data lends itself to cross-validation. We found that the 2 approaches consistently identified several cities with established vector populations at a heightened risk for virus importation, reflecting the potential for spread between countries and cities in Europe. Some of these regions had previously encountered autochthonous transmission (*29*).

The R_0_ estimates, which were derived by using epidemiologic data, were in accordance with the vectorial capacity predictions for the outbreak zones based on local climate conditions. Based on the vectorial capacity, R_0_ can be derived by multiplication with the infectious period. For chikungunya, an infectious period of 3–7 days was reported (*30*). The vectorial capacity of ≈0.7 would give rise to an R_0_ of ≈2–3. This range is within that which we observed in the Rome and Anzio regions in July and August, but the vectorial capacity was estimated to be higher (≈0.8) in the Calabria region, translating into an R_0_ of just over 3–4, which is in agreement with the epidemiologic analysis of the outbreak data (Figure 2).

Although our mobility analysis showed that the local mobility from Var was considerable, no autochthonous chikungunya cases were reported from other identified risk regions along the Mediterranean coast of France and in northern Spain. However, the vectorial capacity of *Ae. albopictus* mosquitoes to transmit the virus is lower in Var than in Lazio, which may explain this discrepancy. Previous studies assessing the risk for local outbreaks after outbreaks outside of Europe found that inbound flight traveler frequencies correlated strikingly well with local reports of virus importation frequencies into Europe (*9*). However, most of these studies evaluated these risks independently and did not attempt to estimate the combined risk for virus importation and climate suitability (*31*,*32*). Moreover, they did not assess local dispersion patterns from airports or outbreak areas. We analyzed big data for long- and short-distance mobility. A major strength of this big data approach is the near real-time availability of mobility patterns based on social media, which are timelier and more accessible and less costly than air passenger data available from commercial providers, such as the IATA. This approach can identify areas of heightened mobility that are potentially at risk for onward transmission, as we have shown in this analysis. Geocoded Twitter data can be a good proxy for human mobility (*15*), but prior research did not explore how such data can be a timely resource for preparedness and response to infectious disease outbreaks.

Similar to others who have used IATA and Twitter data in their studies, we found these novel data sources to be reliable and useful. However, we note that Twitter data can potentially be biased because Twitter users may represent a select population whose mobility patterns differ from those of the general population; more specifically, they represent a population of Twitter users who have allowed Twitter to follow their geolocations. Future studies need to validate the use of social media data in such applications. These methods are an improvement over mobile telephone tracking data because they do not rely on a single provider network and are a less costly data source to acquire.

Seasonal weather forecasts may have provided better input into the assessment of vectorial capacity, specifically for the fall of 2017. Moreover, autochthonous transmission risk may also be related to local proliferation of vectors and local environmental, social, and behavioral characteristics, such as awareness about the symptoms of chikungunya (Appendix 3). Such factors have been found to be associated with the local transmission risk for dengue (*33*). Last, because of the paucity and underreporting of chikungunya cases, we may have potentially underestimated the passenger volume from active transmission areas in Africa.

This study illustrates the potential value of using big data (*18*–*20*) to pinpoint areas at risk for the introduction and dispersion of emerging infectious diseases. The analysis identified that the areas at greatest risk were those in close proximity to the original outbreaks and several larger metropolitan areas. The trajectory and sustained spread of emerging infectious diseases can be anticipated with predictive modeling in realtime. This study suggests that big data can be an indispensable tool for the prevention and control of emerging infectious diseases.

Appendix 1 Detailed methods used in study of big data to monitor the introduction and spread of chikungunya, Europe, 2017.

Appendix 2 Figures demonstrating use of big data to monitor the introduction and spread of chikungunya, Europe, 2017.

Appendix 3 Additional data for study of use of big data to monitor the introduction and spread of chikungunya, Europe, 2017.
