# Supplementary material for: Using Big Data to Monitor the Introduction and Spread of Chikungunya, Europe, 2017
Source: Emerg Infect Dis. 2019 Jun;25(6):1041–9. doi: 10.3201/eid2506.180138 (PMC6537727; doi:10.3201/eid2506.180138)
Supplement: Appendix 1 — Detailed methods used in study of big data to monitor the introduction and spread of chikungunya, Europe, 2017. [file 18-0138-Techapp-s1.pdf]

Article DOI: https://doi.org/10.3201/eid2506.180138

# Using Big Data to Monitor the Introduction and Spread of Chikungunya, Europe, 2017

## Appendix 1

### Epidemic Intelligence Data

We analyzed the 2 outbreak zones in the Var department of France (15 confirmed and 2 probable cases) and around the cities of Anzio and Rome in the Lazio region of central Italy (206 confirmed cases) and 74 confirmed cases in the Calabria region in south Italy (Appendix 3, https://wwwnc.cdc.gov/EID/article/25/6/18-0138-App3.pdf) (*1*–*4*). The disease vector *Ae. albopictus* mosquito is well established in all outbreak regions (*5*). Worldwide monthly chikungunya outbreak reports were compiled by the Epidemic Intelligence team at the European Centre for Disease Prevention and Control (Appendix 3) (*6*). We mapped and visualized the passenger volume of outbound flights to Europe from areas with chikungunya activity by month for March, April, May, and June 2017.

### Air Passenger Volume

We analyzed anonymized flight itinerary data obtained from the IATA Market Intelligence Services and calculated the monthly volume of air passenger-journeys in 2016 (latest data available; presumed to be similar to 2017) from worldwide airports in areas with chikungunya virus active transmission to a final destination in Europe, by using a previously described method (*7*) (Appendix 3). The distribution of the number of passenger-journeys arriving into Europe from airports located in areas with active chikungunya virus transmission was then overlaid with European vector surveillance data compiled by the European Centre for Disease Prevention and Control (VectorNet, https://vectornet.ecdc.europa.eu) for *Ae. albopictus* mosquitoes by using ESRI ArcGIS (*5*).

### Twitter Data

We developed a mining algorithm and collected Tweets by using the Twitter Streaming Application Programming Interface (https://developer.twitter.com). Although the tweets collected from the API represent only ≈1% of the total Tweeter feed, when geographic boundary boxes are used for data collection it provides a high representation of the overall geo-located activity on Twitter (*8*). We filtered the collected tweets based on location by using geocodes, and we extracted only those originating from the study area in July, August, and up to September 19, 2017. We longitudinally analyzed 8,120,417 Tweets. When Tweets from the same users could be followed by geographic coordinates, we obtained users' individual files. We analyzed unidirectional mobility of Twitter users by estimating the frequency of a user being observed in a specific geographic department within the study area and later being observed in any other department within the same month. To compute a rate, we aggregated the total number of movements in a month between any 2 departments and divided this by the total movement across all the departments. The range of all between-department mobility values was 0–1 and added up to 1 when summarized across the departments for inbound and outbound movements. We derived this quantity as a proxy for mobility proximity between any 2 departments and computed it for each month.

### Vectorial Capacity

To estimate seasonal variability in the ability of *Ae. albopictus* mosquitoes to transmit chikungunya virus, we modified our previously established climate dependent vectorial capacity arbovirus models (*9,10*). The model uses temperature and diurnal temperature range to estimate the epidemic potential of an outbreak. Theoretically, vectorial capacity is related to $R_0$. More exactly, the $R_0$ is a function of vectorial capacity (VC) and duration of viremia in humans ($T_h$), that is $R_0 = VC \times T_h$. Vectorial capacity is a function of vector competence, vector lifespan, and extrinsic incubation period (*11*) and is defined mathematically in Appendix 3.

The 4 vector-related parameters in the vectorial capacity are 1) average vector biting rate, $a$; 2) the product of the probability of vector infection ($b_{mi}$) and transmission per bite ($b_{mt}$), $b_m$; 3) extrinsic incubation period, $n$ (i.e., the interval between the acquisition of a pathogen by a vector

and the vector's ability to then transmit the pathogen to another susceptible host); and 4) vector mortality rate, $\mu_m$ ; and 4, female vector-to-human population ratio, *m*.

The effect of temperature on the ability of *Ae. albopictus* mosquitoes to transmit chikungunya virus has not been well studied. However, $\mu_m$ and *a* in relation to temperature have been described for *Ae. albopictus* mosquitoes. We assumed that *n*, $b_m$ would have a dependence on temperature for chikungunya virus transmission similar to that for dengue virus, although we found evidence to support that it can be slightly lower at around 90% (*11–13*) and that *n* is shorter, peaking at around 8 instead of 10 days (*11–13*). Similar to a previous study (*9*), *m* was assumed to be proportional to its temperature-dependent survival curve. Parameter relationships used in the analysis are provided in Appendix 1 Figure.

## Climate Data

We used data from the Climate Research Unit of East Anglia University (*14*) to estimate the average vectorial capacity for July, August, September, and October during 1996–2015. To describe the effect of warmer than usual temperature, we increased the average monthly temperature to its 75th percentile value for each month and recalculated the vectorial capacity. The Climate Research Unit data, originally provided in 0.5° × 0.5° grids by latitude and longitude, were resampled to fit into a grid of 0.01° to better align with the geographic departments of the study area.

### References


1. Italy Ministry of Health. National plan of surveillance and response to arbovirus transmitted by mosquitoes (*aedes* sp.), with particular reference to chikungunya, dengue and zikaviruses—2017 [cited 2019 Apr 3]. http://www.salute.gov.it/portale/temi/documenti/chikungunya/bollettino_chikungunya_20171221.pdf

2. European Centre for Disease Prevention and Control. Cluster of autochthonous chikungunya cases in France–23 August 2017. Stockholm: The Centre; 2017.

3. Venturi G, Di Luca M, Fortuna C, Remoli ME, Riccardo F, Severini F, et al. Detection of a chikungunya outbreak in Central Italy, August to September 2017. Euro Surveill. 2017;22. http://dx.doi.org/10.2807/1560-7917.ES.2017.22.39.17-00646

4. Calba C, Guerbois-Galla M, Franke F, Jeannin C, Auzet-Caillaud M, Grard G, et al. Preliminary report of an autochthonous chikungunya outbreak in France, July to September 2017. Euro Surveill. 2017;22. http://dx.doi.org/10.2807/1560-7917.ES.2017.22.39.17-00647

5. European Centre for Disease Prevention and Control. *Aedes albopictus*—current known distribution in Europe, April 2017. Stockholm: The Centre; 2017.

6. European Centre for Disease Prevention and Control. Communicable disease threats report. Week 26, 25 June–1 July 2017 [cited 2019 Apr 3]. https://ecdc.europa.eu/en/threats-and-outbreaks/reports-and-data/weekly-threats

7. Semenza JC, Sudre B, Miniota J, Rossi M, Hu W, Kossowsky D, et al. International dispersal of dengue through air travel: importation risk for Europe. PLoS Negl Trop Dis. 2014;8:e3278. PubMed http://dx.doi.org/10.1371/journal.pntd.0003278

8. Morstatter F, Pfeffer J, Liu H, Carley KM. Is the sample good enough? Comparing data from Twitter's streaming API with Twitter's Firehose. In: International Conference on Weblogs and Social Media: Association for the Advancement of Artificial Intelligence; 2013. p. 400–8.

9. Rocklöv J, Quam MB, Sudre B, German M, Kraemer MUG, Brady O, et al. Assessing seasonal risks for the introduction and mosquito-borne spread of Zika virus in Europe. EBioMedicine. 2016;9:250–6. PubMed http://dx.doi.org/10.1016/j.ebiom.2016.06.009

10. Liu-Helmersson J. Quam M, Wilder-Smith A, Stenlund H, Ebi K, Massad E, et al. Climate change and *Aedes* vectors: 21st century projections for dengue transmission in Europe. 2016;7:267–77.

11. Christofferson RC, Chisenhall DM, Wearing HJ, Mores CN. Chikungunya viral fitness measures within the vector and subsequent transmission potential. PLoS One. 2014;9:e110538. PubMed http://dx.doi.org/10.1371/journal.pone.0110538

12. Vega-Rua A, Zouache K, Caro V, Diancourt L, Delaunay P, Grandadam M, et al. High efficiency of temperate *Aedes albopictus* to transmit chikungunya and dengue viruses in the southeast of France. PloS One. 2013; 8:e59716. http://dx.doi.org/10.1371/journal.pone.0059716

13. Vega-Rúa A, Zouache K, Girod R, Failloux AB, Lourenço-de-Oliveira R. High level of vector competence of *Aedes aegypti* and *Aedes albopictus* from ten American countries as a crucial factor in the spread of chikungunya virus. J Virol. 2014;88:6294–306. PubMed http://dx.doi.org/10.1128/JVI.00370-14

14. University of East Anglia Climatic Research Unit; Harris IC, Jones PD. CRU TS4.00: Climatic Research Unit (CRU) Time-Series (TS) version 4.00 of high resolution gridded data of month-by-

month variation in climate (Jan. 1901- Dec. 2015). 2017; Centre for Environmental Data Analysis. http://dx.doi.org/10.5285/edf8febfdaad48abb2cbaf7d7e846a86

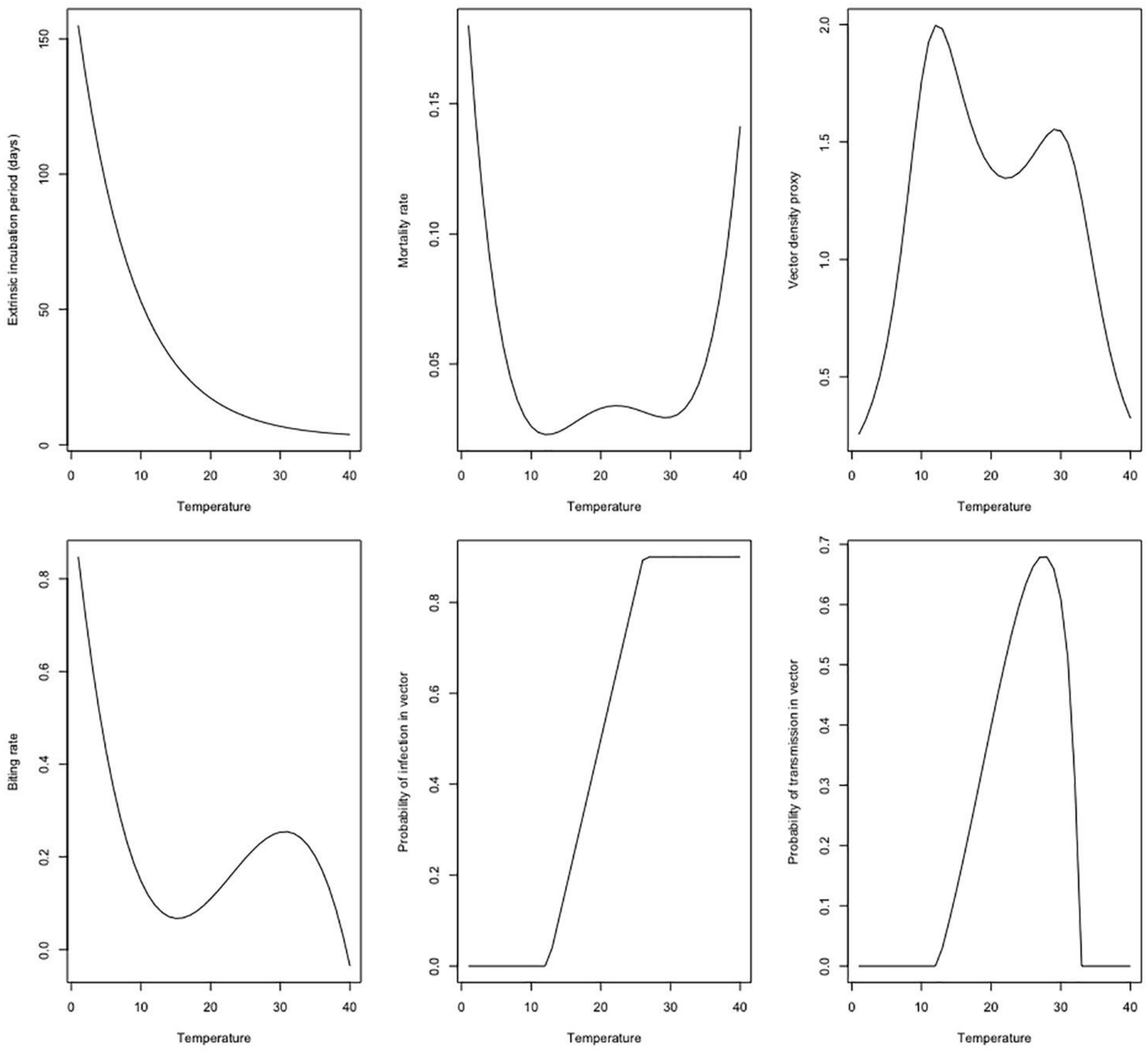


**Appendix 1 Figure**. The relationship of vector-related parameters to temperature (°C), describing the ability of *Aedes albopictus* to transmit chikungunya virus.
