# Supplementary material for: Using Big Data to Monitor the Introduction and Spread of Chikungunya, Europe, 2017
Source: Emerg Infect Dis. 2019 Jun;25(6):1041–9. doi: 10.3201/eid2506.180138 (PMC6537727; doi:10.3201/eid2506.180138)
Supplement: Appendix 2 — Figures demonstrating use of big data to monitor the introduction and spread of chikungunya, Europe, 2017. [file 18-0138-Techapp-s2.pdf]

Article DOI: https://doi.org/10.3201/eid2506.180138

Using Big Data to Monitor the Introduction and Spread of Chikungunya, Europe, 2017

Appendix 2

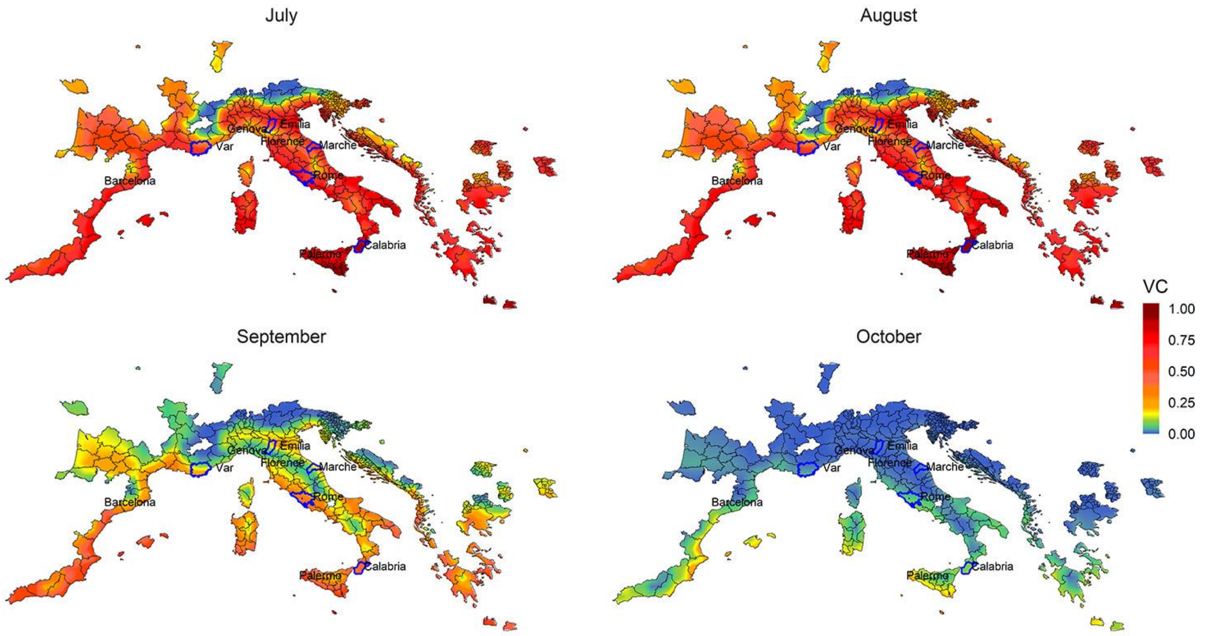

Appendix 2 Figure 1. Average monthly vectorial capacity (VC) estimates derived on the basis of temperature averaging to the 75th percentile of monthly distribution, July-October, 2017. Areas with autochthonous transmission are indicated by colored polygons.

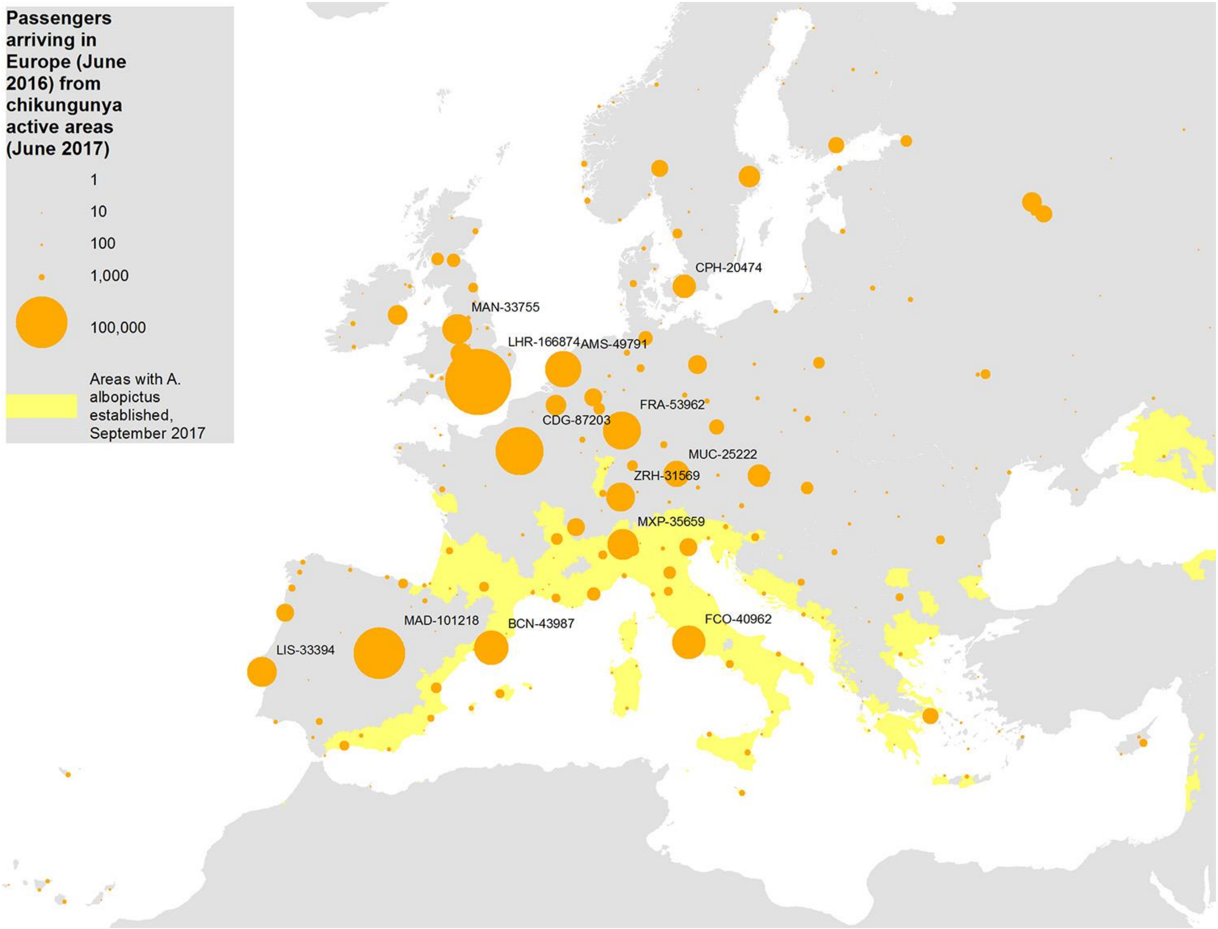

Appendix 2 Figure 2. Number of passengers arriving from chikungunya transmission active areas into Europe, August 2017.

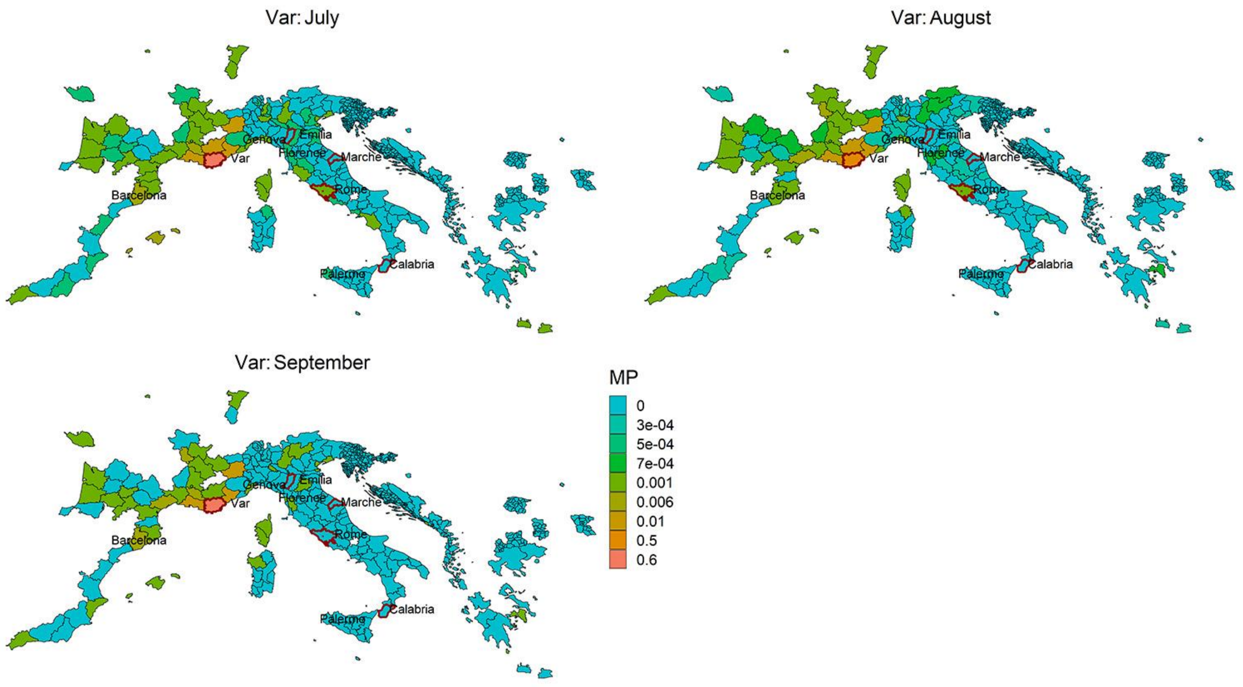

Appendix 2 Figure 3. Mobility proximity (MP) estimates from the Var department, France, to areas in Europe with stable *Ae. albopictus* populations, July-September 2017. The polygons mark the outbreak areas.

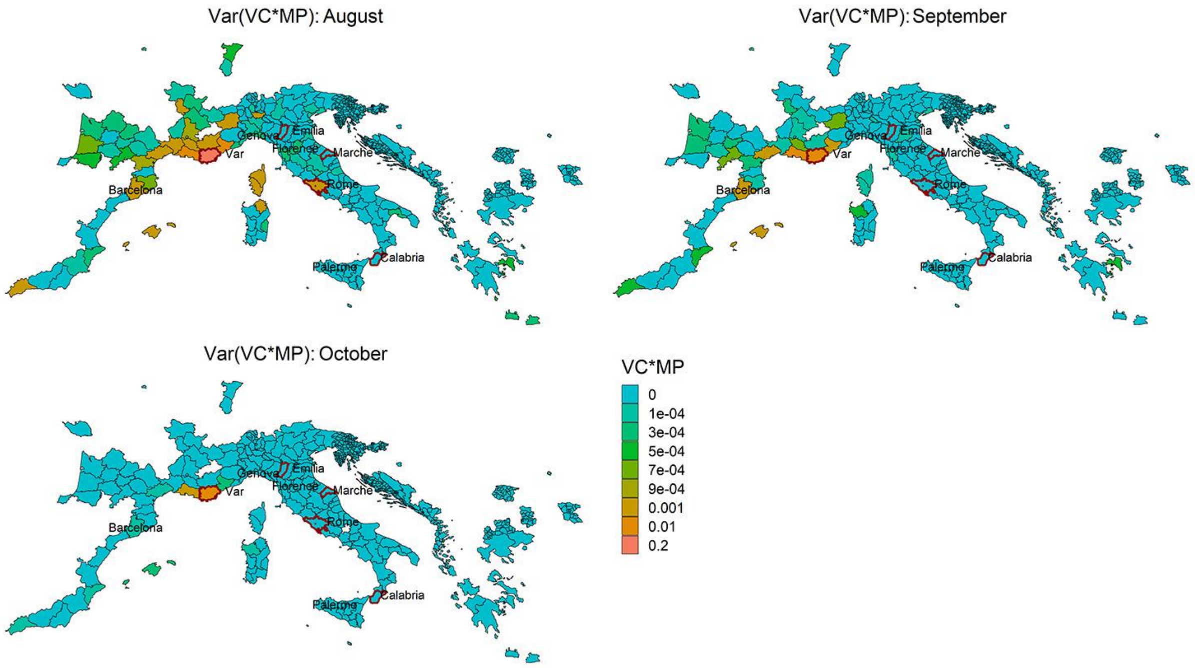

Appendix 2 Figure 4. Estimated risk areas of chikungunya spread from the outbreak areas in the Var department, France, based on combined vectorial capacity (VC) and mobility proximity (MP) estimates, August-October 2017. The polygons mark the outbreak areas.

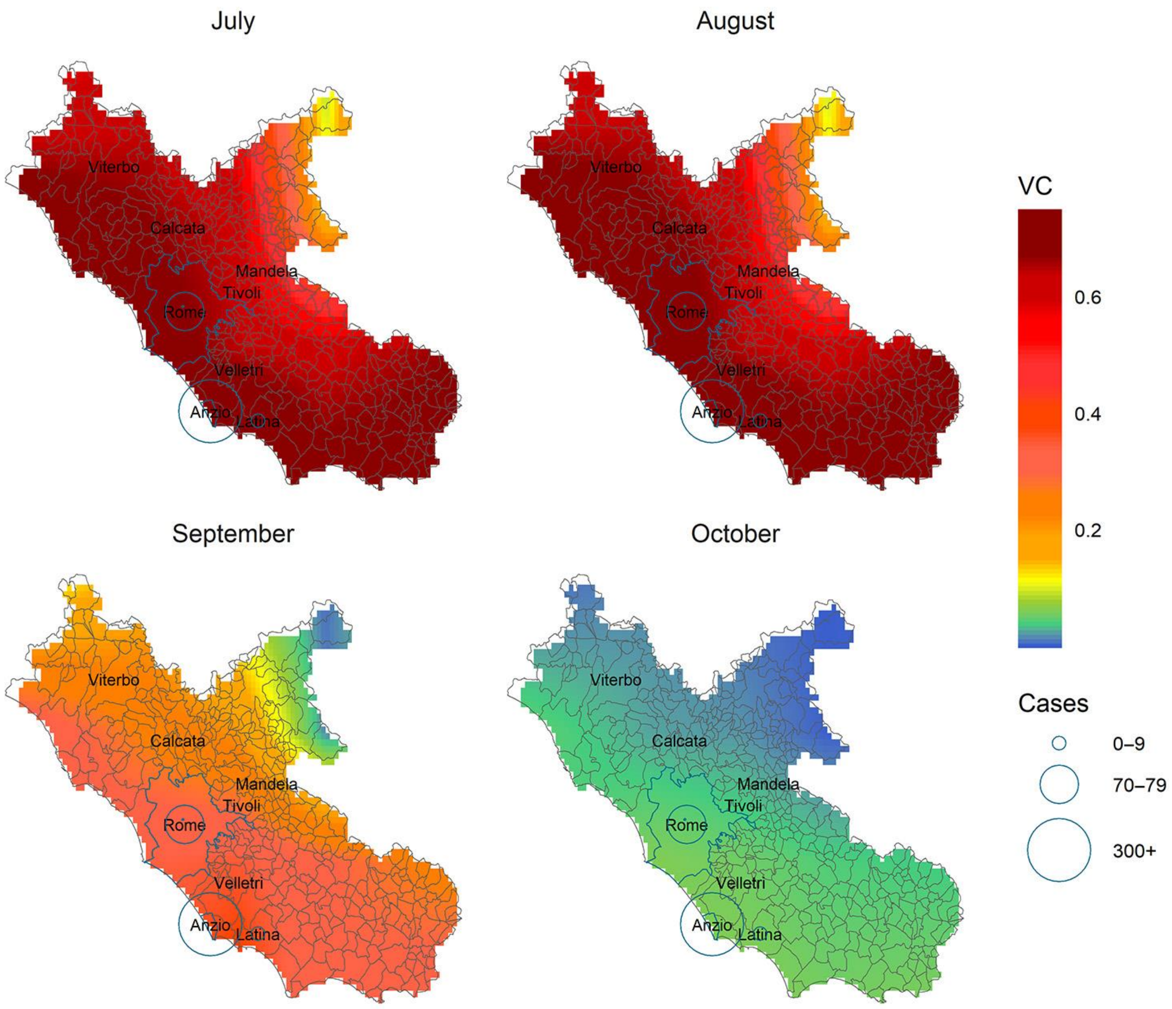

Appendix 2 Figure 5. Vectorial capacity (VC) estimates for Lazio region, July-October 2017, based on average climatic conditions during the period 1996–2015. The number of reported cases are overlaid as circles.

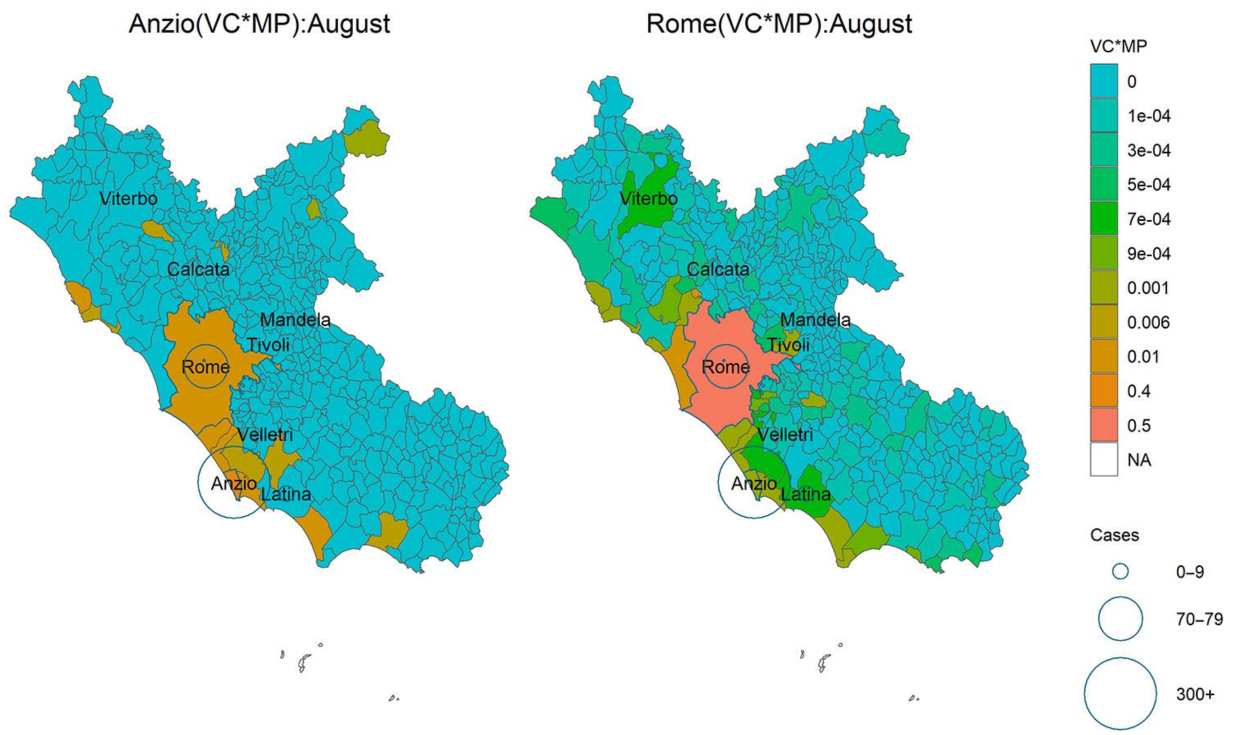

Appendix 2 Figure 6. Estimated risk areas of chikungunya spread from the outbreak areas of Anzio and Rome in Lazio region based on combined vectorial capacity (VC) and mobility proximity (MP) and estimates, August 2017. The number of reported cases are overlaid as circles.

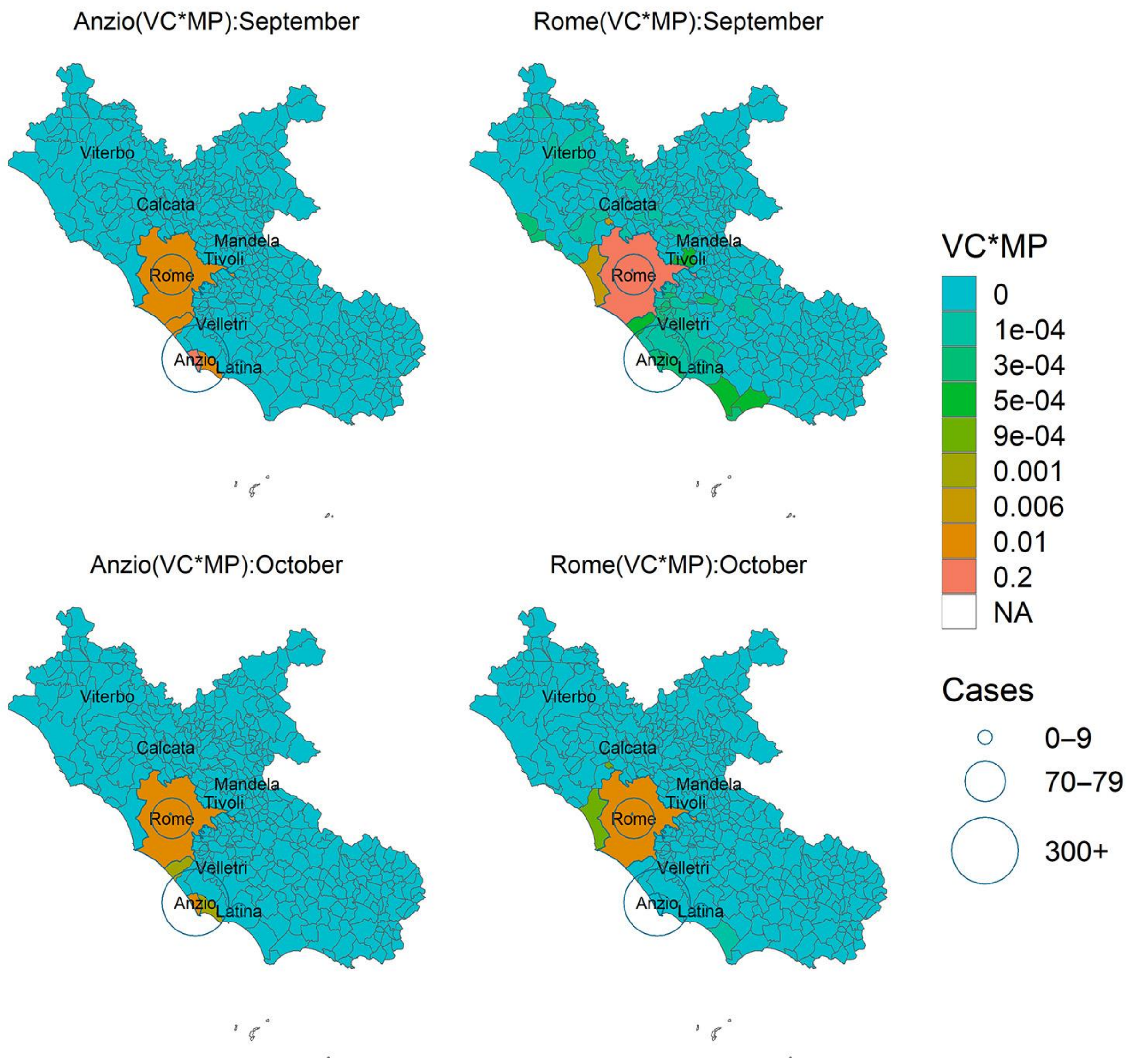

Appendix 2 Figure 7. Estimated risk areas of chikungunya spread from the outbreak areas of Anzio and Rome in Lazio region based on combined vectorial capacity (VC) and mobility proximity (MP) and estimates, September and October 2017. The number of reported cases are overlaid as circles.

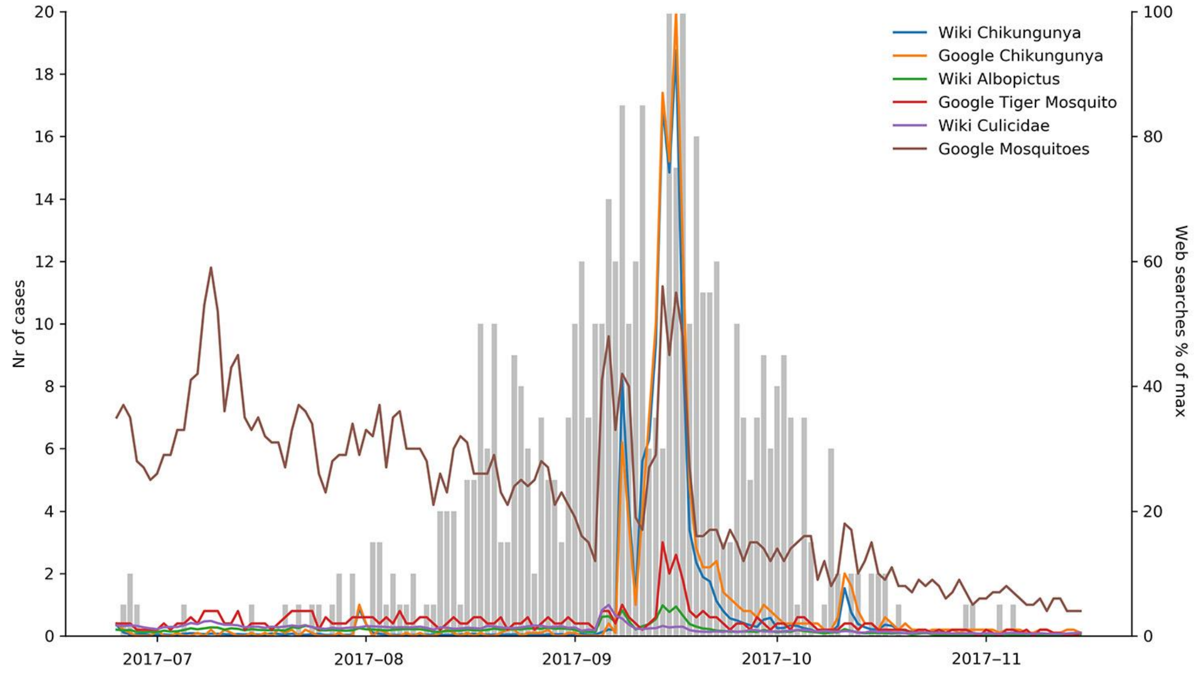

Appendix 2 Figure 8. Hits of Google and Wikipedia on search terms related to mosquito and chikungunya.
