# Supplementary material for: Using Big Data to Monitor the Introduction and Spread of Chikungunya, Europe, 2017
Source: Emerg Infect Dis. 2019 Jun;25(6):1041–9. doi: 10.3201/eid2506.180138 (PMC6537727; doi:10.3201/eid2506.180138)
Supplement: Appendix 3 — Additional data for study of use of big data to monitor the introduction and spread of chikungunya, Europe, 2017. [file 18-0138-Techapp-s3.pdf]

Article DOI: https://doi.org/10.3201/eid2506.180138

Using Big Data to Monitor the Introduction and Spread of Chikungunya, Europe, 2017

Appendix 3

Epidemic Intelligence Data

The analysis was conducted for a specified study area in reasonable proximity to the two outbreak zones in 15 confirmed and 2 probable cases reported in Var department (9 confirmed and 2 probable cases reported in Cannet-des-Maures and 6 confirmed cases in Taradeau) and around the cities of Anzio and Rome in Lazio region of central Italy (206 confirmed cases). Data on confirmed and suspected chikungunya cases were obtained from epidemic intelligence data and reports. The first reports of autochthonous transmission came from Var department, followed by Lazio region, and transmission was reported later on from Calabria (74 confirmed cases), Emilia-Romagna (1 confirmed case) and the Marche (1 confirmed case) regions in Italy (*1*–*4*). In all outbreak regions, the disease vector *Ae. albopictus* is known to be well established (*5*).

Worldwide monthly chikungunya outbreak reports were compiled by the Epidemic Intelligence team at the European Centre for Disease Prevention and Control (ECDC) based on data mining from the World Health Organization, Ministries of Health, and other official and non-official sources, such as media reports, to survey the current worldwide chikungunya situation (*6*). Rather than gauging chikungunya incidence qualitatively, our assessment was based on chikungunya events identified by the ECDC through web crawl searches and from confidential/official sources, such as Early Warning and Response Systems, Program for Monitoring Emerging Diseases, Medical Information System, and Global Public Health Intelligence Network. Weekly notifications from these sources were evaluated and geocoded by month. We mapped and visualized the passenger volume of outbound flights to Europe from areas with chikungunya activity by month, namely for March, April, May, and June 2017.

Air Passenger Volume

The International Air Transport Association (IATA) database has the most voluminous and comprehensive aviation data from over 80,000 travel and online agencies, 400 airlines, and 170 countries. Travelers on commercial, connecting and scheduled charter flights are captured. We analyzed anonymized flight itinerary data obtained from IATA Market Intelligence Services and calculated the monthly volume of air passenger-journeys in 2016 (latest data available; presumed to be similar to 2017) from airports worldwide located in areas with chikungunya active transmission with a final destination in Europe. We assumed that human passenger-journeys were the main vehicle of viral spread, rather than infected mosquitoes in airplanes, based on the index cases of past outbreaks that had a travel history to endemic areas. These large-scale IATA passenger data represent ≈93% of the world's commercial air traffic, while the remainder was estimated using market intelligence. The distribution of number of passenger-journeys arriving into Europe from airports located in areas with active chikungunya transmission was then overlaid with European vector surveillance data compiled by the ECDC (VectorNet) for *Ae. albopictus* using ESRI ArcGIS (*5*). Chikungunya continues to spread internationally due to several factors, most notably the adaptive mutations in the viral genome that enabled the virus to be more easily transmitted by *Ae. albopictus* (*7*). This vector has expanded its geographic range through increasing global trade of used tires and plants and established itself in areas with suitable climate and habitat in many parts of the world. However, *Ae. aegypti*, another competent vector for the Italian 2017 chikungunya virus strain (*8*), is largely not present in continental Europe with the exception of a small region around the eastern coast of the Black Sea. Once an outbreak occurs the disease can entrench itself in the local vector population and become endemic if climate allows vectors to be active around the year.

Vectorial Capacity

The vectorial capacity can be described by the following mathematical expression:

$VC = ma^2 b_m e^{-\mu mn}/\mu m$. See Appendix 1, https://wwwnc.cdc.gov/EID/article/25/6/18-0138-App1.pdf, for a more detailed description.

Wikipedia and Google Trend Data

Wikipedia is a free, internet-based encyclopedia structured as an interconnected network of open-content articles and is considered one of the top Web sites visited globally (*9*). Internet users typically use Wikipedia to access background information on a specific topic and related subtopics. Although web searches, usually using Google, lead users to a Wikipedia article, the majority of users follow the links provided in the article to access other related articles. Therefore, it has been argued that Wikipedia access statistics may provide valuable insight into the emergence and shift of collective interests or activities of individuals, and sudden peaks in user access of specific Wikipedia pages may reflect extreme events in nature or society (*10*). Here we chose specific articles related, namely mosquitoes, albopictus and chikungunya, across the Italian, French, German (as control) and English (as reference) language editions of Wikipedia, and extracted daily article access logs, which provide a summary file listing the number of access requests for each article per day in each language during the period from July to November 2017. The Wikipedia data was downloaded 2018-10-13 using the mwviews.api/PageviewsClient (https://github.com/mediawiki-utilities/python-mwviews) Wikipedia articles "Aedes_albopictus" (redirected from "Zanzara tigre" = tiger mosquito), "Culicidae" (redirected from "Zanzara/e" = mosquitoes singular and plural), "Chikungunya) We also downloaded Google Trends data 2018-01-26 from https://trends.google.com/trends/explore?date = 2017-06-25%202017-11-15&geo = IT&q = %2Fm%2F09f96,%2Fm%2F01__7l,%2Fm%2F01yy_q using search topics which include several similar search terms (https://support.google.com/trends/answer/4359550). As Wikipedia gives absolute page hits and Google gives only proportions, the Wikipedia articles were added and calculated as percentages from the maximum number of page hits of the three search terms.

Our analysis of Wikipedia access logs and Google Trends shows clear peaks in terms of number of access requests for the articles on mosquitoes (Culicidae) and Albopictus first in June/July and then in mid-September in the Italian language version of Wikipedia (Appendix 2 Figure 8, https://wwwnc.cdc.gov/EID/article/25/6/18-0138-App2.pdf). A distinct peak in access requests was also observed for Tiger mosquitoes in mid-September in Italian language. We did not observe such peaks for these Wikipedia articles in 2016. For the articles on chikungunya, we

observed peaks in early August in Italian Wikipedia and in mid-August in French Wikipedia, which probably indicates an increasing awareness of the disease among the public. We observed a larger peak in access requests on chikungunya in Italian Wikipedia later in mid-September, followed by another small peak in mid-October, probably as a result of the continued exposure of the public through the media to the outbreak news because of its spread. We found a strong correlation between the number of notified chikungunya cases and the access requests for chikungunya in the Italian language version of Wikipedia (Figure 2). An overlay with the search data on chikungunya from Google yielded a similar pattern, probably because Wikipedia hits are typically preceded by Google searches (Appendix 2 Figure 8). These observations suggest that Wikipedia access logs to articles on specific health topics have the potential to supplement disease surveillance and outbreak prediction efforts when combined with disease incidence data, as demonstrated in (*11*).

References

1. European Centre for Disease Prevention and Control. Cluster of autochthonous chikungunya cases in France–23 August 2017 [cited 2019 Apr 3]. https://ecdc.europa.eu/sites/portal/files/documents/RRA-Chikungunya-France-revised-Aug-2017.pdf

2 Venturi G, Di Luca M, Fortuna C, Remoli ME, Riccardo F, Severini F, et al. Detection of a chikungunya outbreak in central Italy, August to September 2017. Euro Surveill. 2017;22(39):pii :17-00646. http://dx.doi.org/10.2807/1560-7917.ES.2017.22.39.17-00646

3. Italy Ministry of Health. National plan of surveillance and response to arbovirus transmitted by mosquitoes (*aedes* sp.), with particular reference to chikungunya, dengue and zikaviruses–2017 [cited 2019 Apr 3]. http://www.salute.gov.it/portale/temi/documenti/chikungunya/bollettino_chikungunya_20171221.pdf

4. Calba C, Guerbois-Galla M, Franke F, Jeannin C, Auzet-Caillaud M, Grard G, et al. Preliminary report of an autochthonous chikungunya outbreak in France, July to September 2017. Euro Surveill. 2017;22(39). http://dx.doi.org/10.2807/1560-7917.ES.2017.22.39.17-00647

5. European Centre for Disease Control and Prevention. *Aedes albopictus*–current known distribution in Europe, April 2017 [cited 2019 Apr 3]. https://ecdc.europa.eu/en/publications-data/aedes-albopictus-current-known-distribution-europe-april-2017

6. European Centre for Disease Prevention and Control. Communicable disease threats report. Week 26, 25 June–1 July 2017 [cited 2019 Apr 3]. https://ecdc.europa.eu/en/threats-and-outbreaks/reports-and-data/weekly-threats

7. Tsetsarkin KA, Weaver SC. Sequential adaptive mutations enhance efficient vector switching by chikungunya virus and its epidemic emergence. PLoS Pathog. 2011;7:e1002412. PubMed http://dx.doi.org/10.1371/journal.ppat.1002412

8. Carletti F, Marsella P, Colavita F, Meschi S, Lalle E, Bordi L, et al. Full-length genome sequence of a chikungunya virus isolate from the 2017 autochthonous outbreak, Lazio region, Italy. Genome Announc. 2017;5:e01306-17. PubMed http://dx.doi.org/10.1128/genomeA.01306-17

9. Schroeder R, Taylor L. Big data and Wikipedia research: social science knowledge across disciplinary divides. Inf Commun Soc. 2015;18:1039–56. http://dx.doi.org/10.1080/1369118X.2015.1008538

10. Kämpf M, Tismer S, Kantelhardt JW, Muchnik L. Fluctuations in Wikipedia access-rate and edit-event data. Physica A. 2012;391:6101–11. http://dx.doi.org/10.1016/j.physa.2012.07.004

11. Generous N, Fairchild G, Deshpande A, Del Valle SY, Priedhorsky R. Global disease monitoring and forecasting with Wikipedia. PLOS Comput Biol. 2014;10:e1003892. PubMed http://dx.doi.org/10.1371/journal.pcbi.1003892
